# Effects of Nutrient Intake on Diagnostic Measures of Sarcopenia among Arab Men: A Cross-Sectional Study

**DOI:** 10.3390/nu13010114

**Published:** 2020-12-30

**Authors:** Maha H. Alhussain, Shaea Alkahtani, Osama Aljuhani, Syed Shahid Habib

**Affiliations:** 1 Department of Food Science and Nutrition, College of Food and Agriculture Sciences, King Saud University, Riyadh 11451, Saudi Arabia; 2 Department of Exercise Physiology, College of Sport Sciences and Physical Activity, King Saud University, Riyadh 11451, Saudi Arabia; shalkahtani@ksu.edu.sa; 3 Department of Physical Education, College of Sport Sciences and Physical Activity, King Saud University, Riyadh 11451, Saudi Arabia; oaljuhani@ksu.edu.sa; 4 Department of Physiology, College of Medicine, King Saud University, Riyadh 11451, Saudi Arabia; sshahid@ksu.edu.sa

**Keywords:** sarcopenia, muscle mass, muscle strength, nutrition, food frequency questionnaire

## Abstract

Sarcopenia is a major public health condition and is, therefore, of great clinical interest. However, the role of nutrient intake in sarcopenia is unclear. We examined the associations between nutrient intake and diagnostic measures of sarcopenia, including low muscle mass (appendicular lean mass (ALM) divided by height squared, ALM/h^2^) and strength (hand-grip strength, HGS) among Arab men. This cross-sectional study included 441 men aged 46.8 ± 15.98 years. Habitual nutrient intake was assessed using a food frequency questionnaire (FFQ). Participants were classified according to different ALM/h^2^ and HGS reference values. Participants with normal muscle mass, defined by an ALM/h^2^ cutoff of <8.68 kg/m^2^ (−1 standard deviation (SD) <reference values Arab men), had greater daily energy, protein and fat intake, and percentage of energy from protein and fat (*p* < 0.01). Conversely, normal muscle mass was associated with a lower percentage of energy from carbohydrates (CHO) (*p* < 0.001). Regarding muscle strength, participants with HGS above 42 kg (median HGS of Arab men) had higher daily energy and protein and fat intake, but a lower percentage of energy from CHO and a lower intake of total omega-3 fatty acids (*p* < 0.05). Individuals with normal muscle mass and high HGS have greater daily energy, protein, and fat intake and a lower percentage of energy from CHO compared to sarcopenic individuals.

## 1. Introduction

Aging is an inevitable phenomenon associated with progressive changes in body composition and a gradual decline in muscle mass [1,2]. The term sarcopenia, first introduced by Rosenberg, is used to describe this age-related loss of muscle mass [3]. In 2010, the European Working Group on Sarcopenia in Older People (EWGSOP) developed a consensus set of diagnostic criteria for sarcopenia based on low muscle mass, determined by appendicular lean mass (ALM), combined with low muscle function, determined by handgrip strength (HGS) and/or gait speed. The EWGSOP also recommended using appropriate measurements and cutoff points as references for sarcopenia diagnosis [4]. In 2018, the Second European Working Group on Sarcopenia in Older People (EWGSOP2) [5] revised the original recommendations and used low muscle strength as the primary diagnostic measure of sarcopenia. Low muscle strength is better than mass at predicting adverse health outcomes [6,7,8].

Although overt sarcopenia is more pronounced in older adults, the development of sarcopenia begins during middle age (40–50 years old) in some individuals [9]. Sarcopenia is of great clinical interest because it predicts frailty, disability, decreased quality of life, loss of independent living, and mortality [10,11,12]. Furthermore, at the societal level, sarcopenia leads to increased health care costs [13]. Accordingly, the need to understand more about its etiology is of immediate interest. The onset and progression of sarcopenia can be accelerated by numerous factors, including chronic diseases and lifestyle behaviors like physical inactivity and poor diet [14]. Physical activity is often cited as a modifiable lifestyle factor that is negatively associated with low muscle mass and strength [15,16]. However, the role of nutrient intake in sarcopenia has not been thoroughly investigated. Several studies link adequate intake of single nutrients, such as protein and omega-3 fatty acids, to improvements in muscle mass and strength in older adults [17,18,19]. Micronutrients have also been linked to muscle mass and strength, including vitamin D, vitamin E, calcium (Ca), and magnesium (Mg) [20,21,22,23,24,25,26]. In addition to inadequate intake of certain nutrients, the total energy intake and percentage contribution of energy from macronutrients may influence the risk for sarcopenia. A study by Park et al. [20] reported that sarcopenia is inversely correlated with total energy, protein, and carbohydrates (CHO) intake in older adults. Another recent study by Beaudart et al. [26] concluded that sarcopenic individuals seem to consume significantly reduced amounts of total energy, proteins, and fat compared with nonsarcopenic individuals.

Few population-based studies have investigated the association between nutrient intake and diagnostic measures of sarcopenia [27]. To address this gap in extant literature, more investigation is required to enhance the evidence for dietary recommendations to prevent or delay the development of sarcopenia. Thus, the primary aim of this study was to examine the associations between dietary nutrient intake and diagnostic measures of sarcopenia, muscle mass (ALM by bio-impedance analyzer (BIA)) and muscle strength (HGS), in Arab men in Riyadh, Saudi Arabia. The associations of anthropometry and body composition with muscle mass and strength were also assessed.

## 2. Materials and Methods

### 2.1. Participants

A total of 441 participants were recruited via a poster advertisement sent to Community Development Commissions of Riyadh districts and posted on social media platforms. Inclusion requirements included male sex, aged 18–85 years, BMI ≤ 40 kg/m^2^, Saudi or Arab Riyadh residents, and the ability to move independently. Exclusion criteria included professional athletes, cardiovascular diseases, musculoskeletal diseases, cerebrovascular accidents, chronic obstructive pulmonary disease, cancer, and dementia. Using G*Power [28] calculation, power analysis was calculated for actual power = 0.948 using the effect size of HGS = 0.75.

The study was conducted according to the guidelines established by the Declaration of Helsinki and the study was approved by the institutional review board (IRB) of King Saud University (IRB No. E-18-3381). Informed written consent was obtained from all participants before study enrollment.

### 2.2. Study Design

The community-based, descriptive, cross-sectional study was conducted between October 2018 and June 2019. After an initial telephone interview, potentially eligible participants were invited to selected sites for data collection. Data were collected at different sites depending on the participant age. For men aged under 40 years, data were collected at the Exercise Physiology Unit, College of Sport Sciences and Physical Activity, King Saud University, Riyadh, Saudi Arabia. For men aged 40 and over, data were collected at seven Community Development Commissions located in the north, south, east, and central districts of Riyadh. Participants arrived early in the morning (~8:00 A.M.) after fasting overnight (at least 8 h) and were given detailed information about the study. Eligible participants had anthropometry and body composition measured. They were then asked to complete a validated food frequency questionnaire (FFQ) to assess their typical food intake.

### 2.3. Measurements

#### 2.3.1. Anthropometry

Weight, height, and waist circumference (WC) were measured using standardized protocols [29]. Weight was measured to the nearest 0.1 kg using a calibrated, digital scale (PD100 ProDoc, Detecto Scale, Cardinal, Webb City, MO, USA); participants wore light clothing and no shoes. Height was measured to the nearest 0.1 cm using a stadiometer (Seca 213, Seca GmbH & Co., Hamburg, Germany) without shoes and with participants in a freestanding position. WC was measured to the nearest 0.1 cm in a horizontal plane at the level of the midpoint between the lower margin of the last rib and the crest of the ileum when the subject stood with feet 25–30 cm apart using a flexible non-stretch tape. Body mass index (BMI) was calculated (weight (kg)/height^2^ (m)).

#### 2.3.2. Body Composition

Body composition was assessed using a multi-frequency Tanita MC-980MA BIA (Tanita Corporation, Tokyo, Japan), according to the manufacturer’s guidelines. This analyzer was designed to measure the body composition in segmental parts of the whole body, including arms, legs, and the trunk area. The measurement procedure requires the subject to stand in bare feet on the metal plates of the analyzer and hold a pair of handgrips, one in each hand. The bio-impedance component of the measurements took approximately 30 seconds per participant and output was printed. Absolute body fat, body fat %, and muscle mass were obtained from these analyses.

#### 2.3.3. Diagnostic Measures of Sarcopenia

##### Muscle Mass

ALM was used to calculate the ratio of total lean arm and leg mass [30] to the squared height (ALM/h^2^). Based on EWGSOP2 recommendations [5], muscle mass was considered low if ALM/h^2^ < 7.0 kg/m^2^ which indicates a predictor of sarcopenia. A local cutoff value for ALM/h^2^ (−1 standard deviation (SD) <reference values young Arab men; 8.68 kg/m^2^) [31] was also used in this study to identify people with low muscle mass.

##### Muscle Strength

The muscle strength was measured using a handgrip test. HGS (maximum voluntary contractions) was measured twice using a standardized protocol [32] with a manual spring dynamometer (Baseline® Smedley Spring Dynamometers, Fabrication enterprises Inc., NY, USA). Participants were instructed to hold the dynamometer in their dominant hand with the arm stretched parallel to the body while standing upright. The best performance (in kg) was considered the maximum grip strength used for further analysis.

Based on HGS values, participants were divided into three groups: a low HGS group (<27 kg; EWGSOP2 cutoff [5]); a second group with HGS > 42 kg, which was the median HGS of 471 Arab men [31]; and a third group with HGS ranging from 27 to 42 kg, which is higher than the risk of low HGS (≥27 kg) but < 50% in the same population (≤42 kg).

#### 2.3.4. Nutrient Intake Assessments

Dietary intake was assessed using a self-administered FFQ, designed and validated to measure participants’ habitual diet over the previous 12 months [33]. The questionnaire was developed in the Arabic language. A list of 140 common Saudi food items was included in the questionnaire, where a closed-ended approach was used. Participants were asked to indicate the average consumption frequency of each FFQ item using nine frequency categories as follows: never or less than once a month, 1–3 per month, once a week, 2–4 per week, 5–6 per week, once a day, 2–3 per day, 4–5 per day, and more than 6 per day. The portion sizes were described and supported by household measures. Participants completed the FFQ in paper or electronic format and a trained dietitian reviewed the questionnaire. Exclusions were made due to invalid or incompletely filled FFQs.

Nutritional intakes of energy, CHO, protein, fat, total omega-3 fatty acids (sum of a-linolenic acid (ALA), eicosapentaenoic acid (EPA), docosapentaenoic acid (DPA), and docosahexaenoic acid (DHA)), vitamin D, vitamin E, Ca, and Mg were assessed. These nutrients were chosen based on previous studies demonstrating associations between these nutrients and sarcopenia [34,35,36,37,38]. Average daily energy and nutrient intakes were calculated using the U.S. Department of Agriculture (USDA) software (Edition 27, 2014, Beltsville, MD, USA) and the Nutribase software (Edition 11, 2014, CyberSoft, Inc, Phoenix, AZ, USA), which utilizes food macronutrients and micronutrients. Additionally, for Saudi traditional food, an Arabic food analysis program was used (1st version, 2007).

### 2.4. Statistical Analysis

All statistical tests were completed using IBM SPSS Statistics version 23 (IBM Corp., Armonk, NY, USA). Data were checked for normality using the Kolmogorov–Smirnov test and the appropriate transformations were applied for non-normally distributed data. Data are presented as a mean ± SD for normally distributed variables and median (Q1–Q3) for non-normally distributed variables. Independent *t*-test and Mann–Whitney U test were used to compare mean or median differences in normal and low ALM/h^2^ groups.

One-way ANOVA was used to compare mean differences in HGS groups. When an overall statistically significant difference in group means was shown, Tukey’s post hoc test was performed to confirm where the differences occurred between groups. Pearson correlation coefficient analyses were performed to study the relationships between ALM/h^2^ or HGS and each parameter. Correlations were classified as weak if *r* < 0.5, moderate if *r* ≥ 0.5 to < 0.8, strong if *r* ≥ 0.8, and perfect if *r* = 1 [39]. Multiple regressions were used to calculate the correlation between ALM/h^2^ or HGS and each category (age and anthropometry (height, body weight, BMI and WC), body composition (body fat, fat mass, and muscle mass), and nutrient intake (energy, CHO [%], CHO [g/day], protein [%], protein [g/day], fat [%], fat [g/day], total omega-3 fatty acids, vitamin D, vitamin E, Ca, and Mg)) and to calculate R^2^ to study the effect of each category on ALM/h^2^ or HGS. Stepwise procedures were used in the multiple regression analysis to determine the significant variables that affect ALM/h^2^ or HGS in each category. Age-specific subgroup sensitivity analyses were performed for both ALM/h^2^ and HGS for participants aged over 65 years. Statistical significance was set at <0.05 for all statistical tests.

## 3. Results

### 3.1. Participant Characteristics

**Figure 1:**
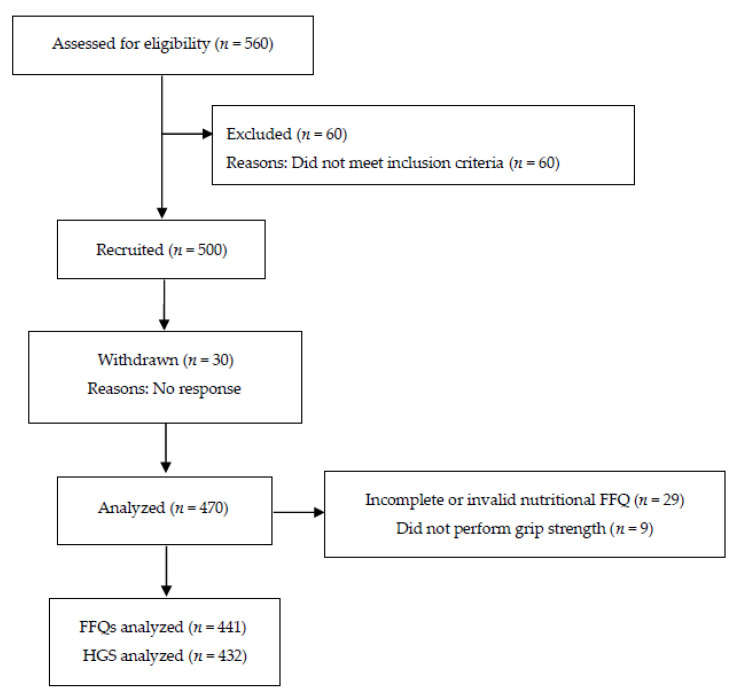
Flow chart of enrollment for the study.

Of the 500 participants enrolled in the study, 30 participants withdrew (no longer interested in study participation), 470 participants completed the study, 29 participants were excluded due to incomplete or invalid nutritional data (FFQ), and 441 participants were included in the final analysis. Participants who were missing values for HGS were excluded only from the HGS comparisons (Figure 1). General characteristics and nutrient intake of the study participants are summarized in Table 1. 

### 3.2. Between-Group Differences

Table 2 displays the differences between the normal and low ALM/h^2^ groups (mean ± SD ALM/h^2^: 8.98 ± 1.21 kg/m^2^ and 6.37 ± 0.69 kg/m^2^, respectively) with regard to general characteristics and nutrient intake. Using the EWGSOP2 cutoff, low muscle mass was observed in 4.8% of the participants. Participants with low muscle mass were significantly older and had lower BMI, lower fat mass, lower muscle mass, and lower HGS (*t*-test, *p* < 0.05). No other significant differences were observed between participants with low muscle mass and those with normal muscle mass, including nutrient intake. According to the local cutoff, participants with low muscle mass (46% of the participants) were older, taller, and had lower BMI, WC, body fat %, muscle mass, and HGS (*t*-test, *p* < 0.01). In terms of nutrient intake, those with low muscle mass differed in their total energy intake, CHO (as a percentage of energy intake; energy%), protein (g/day), protein (energy%), fat (g/day), and fat (energy%) compared with those with normal muscle mass (*t*-test, *p* < 0.05). However, no significant differences were observed in other nutrient intakes.

The general characteristics and nutrient intake of participants with and without low muscle strength according to HGS using different reference values are shown in Table 3. The percentages of participants who had HGS < 27 kg, 27–42 kg, and above 42 kg were 8.1% (mean ± SD HGS: 22.31 ± 3.49 kg), 54.7% (mean ± SD HGS: 35.70 ± 4.13 kg), and 37% (mean ± SD HGS: 48.37 ± 4.62 kg), respectively. There were significant differences between the three groups with respect to age, height, body weight, BMI, muscle mass, and ALM/h^2^ (one-way ANOVA, *p* < 0.01). As shown in Table 3, participants with HGS < 27 kg were the oldest and shortest and had the lowest body weight, muscle mass, and ALM/h^2^. Regarding nutrient intake, there were significant differences between the three groups in total energy intake, CHO (energy%), protein (g/day), fat (g/day), and total omega-3 fatty acids (one-way ANOVA, p < 0.05). Participants who had HGS > 42 kg had higher total energy, protein (g/day), and fat (g/day) intakes compared with participants with HGS between 27 and 42 kg. Additionally, the highest intake of total omega-3 fatty acids was observed in participants with HGS < 27 kg. There were no significant differences among the three groups in micronutrient intake.

### 3.3. Correlation Analysis

**Figure 2:**
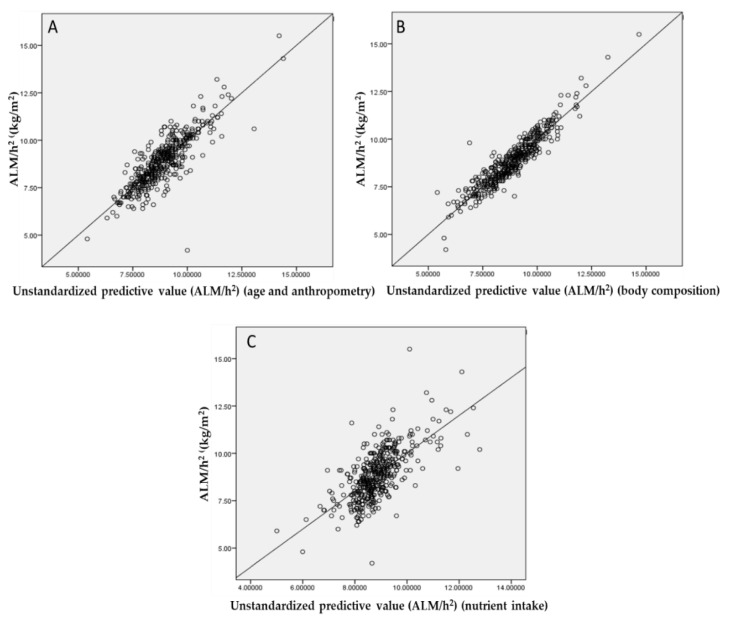
Multiple regression analyses between ALM/h^2^ and age and anthropometry ((**A**) R^2^ = 0.869), body composition ((**B**) R^2^ = 0.713), and nutrient intake ((**C**) R^2^ = 0.450). (*n* = 441). Abbreviations: ALM, appendicular lean mass.

Pearson’s correlations between ALM/h^2^ and HGS and each parameter are shown in Table 4. Significant positive correlations were observed between ALM/h^2^ and anthropometry, body composition, protein (g/day), protein (energy%), and fat (g/day). ALM/h^2^ and muscle mass had the strongest correlation (strong correlation). Significant negative correlations were also observed between ALM/h^2^ and age and CHO (energy%). Conversely, no significant correlations were found between ALM/h^2^ and CHO (g/day), fat (energy%), and micronutrients. Multiple regression analyses (Figure 2) showed that body composition explained 86.9% (R^2^) of the variance in ALM/h^2^. Muscle mass and body fat percentage contributed to this variance, as determined by a stepwise procedure. Age and anthropometric measurements explained 71.3% (R^2^) of the variance in ALM/h^2^, while dietary intake explained 45.0% (R^2^) of the variance in ALM/h^2^. Age, BMI, WC, body weight, energy (kcal/day), CHO (energy%), protein (g/day), and fat (energy%) contributed to this variance. Age-specific subgroup sensitivity analyses were performed for ALM/h^2^ for participants aged over 65 years and RUC = 0.730 ((0.664–0.796) 95%CI, (*p* < 0.001)) was observed.

**Figure 3:**
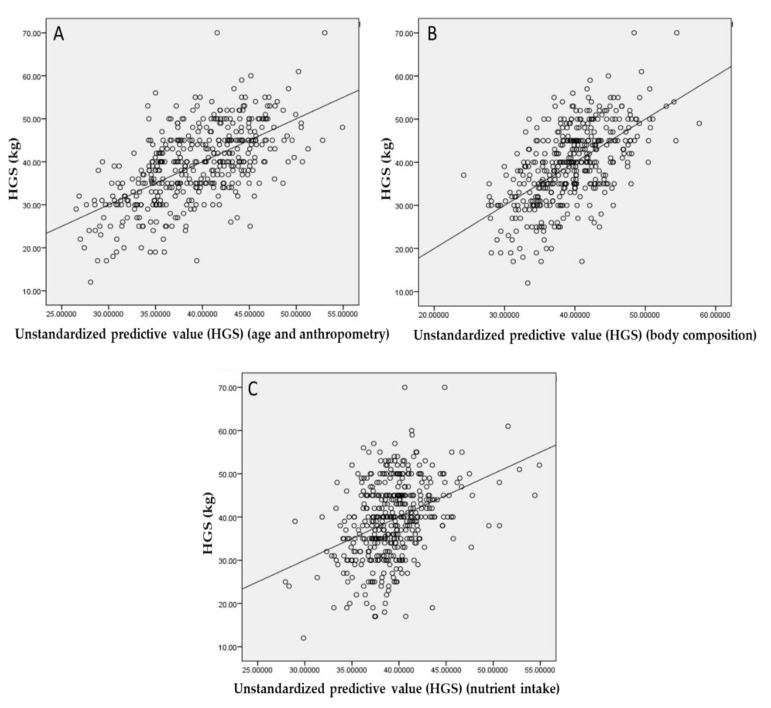
Multiple regression analyses between HGS and age and anthropometry ((**A**) R^2^ = 0.360), body composition ((**B**) R^2^ = 0.347), and nutrient intake ((**C**) R^2^ = 0.146). (*n*= 432). Abbreviations: HGS, handgrip strength.

HGS was positively correlated with height, weight, BMI, fat mass, muscle mass, energy (kcal/day), protein (g/day), and protein (energy%). However, these correlations were weak and the only moderate correlation was observed between HGS and muscle mass. Weak negative correlations were found between HGS and age, body fat%, CHO (energy%), and total omega-3 fatty acids. HGS did not significantly correlate with the other parameters. According to a multiple regression analysis (Figure 3), age and anthropometric measurements accounted for 36% of the HGS variance. The results from the stepwise regression analysis revealed that age, body weight, and height contributed to this variance. Body composition with contribution of body fat percentage accounted for 34.7% (R^2^) of the HGS variance. Nutrient intake contributed to 14.6% (R^2^) of the variance in HGS, and energy (kcal/day), CHO (energy%), and fat (g/day) contributed to this variance. Age-specific subgroup sensitivity analyses were performed for HGS for participants aged over 65 years; RUC = 0.839 ((0.0.783–0.895) 95%CI, *p* < 0.001)) was detected.

## 4. Discussion

In the present study, we investigated the associations between muscle mass and strength with dietary nutrient intake, anthropometry, and body composition in Arab men. Participants were defined as having low muscle mass and strength using international (EWGSOP2) and local cutoffs for ALM/h^2^ and HGS. Our results showed that participants with normal muscle mass, as defined by a local cutoff of ALM/h^2^, had greater daily energy, protein, and fat intakes (g/day) and that a greater percentage of their energy came from protein and fat (energy%). Conversely, normal muscle mass was associated with a lower percentage of energy from CHO (energy%). Regarding muscle strength, participants with HGS above 42 kg had higher daily energy, protein, and fat intakes (g/day). On the other hand, a lower percentage of energy from CHO (energy%) was found in participants with HGS above 42 kg and, surprisingly, these participants had a lower intake of total omega-3 fatty acids (g/day).

In agreement with our energy intake findings, several studies have demonstrated that energy intake is associated with sarcopenia and muscle mass [40,41]. Reduced energy intake causes a reduction in protein synthesis [42]. The differences in energy intake in the present study can be explained by the significantly younger age of participants with higher ALM/h^2^ and HGS. A gradual decline in energy intake in old age has been reported [43,44,45]. This can be caused by aging processes, including physiological changes, the presence of diseases, and the use of medications. Age-related decline in energy intake is accompanied by a reduction in the percentage of energy from fat, whereas the contribution of CHO to energy intake increases [45,46]. In the present study, similar results were observed in ALM/h^2^ groups when the local cutoff of ALM/h^2^ was used. Additionally, the contribution of CHO to energy intake was the lowest in participants with HGS above 42 kg. These findings suggest that the percentage of energy from macronutrients may have important effects on muscle mass and strength maintenance with advancing age.

Increasing protein intake has been proposed as an important pillar of sarcopenia treatment [47,48]. Insufficient protein intake can contribute to loss of muscle mass and strength due to chronic disruption in the balance between muscle protein synthesis and degradation [49]. In the current study, a significant difference in protein intake (g/day) was observed within muscle mass and strength groups. Comparable results were found in previous studies [26,50] in which a lower intake of protein was reported in sarcopenic versus nonsarcopenic older adults. We also observed a greater intake of fat (g/day) in groups with normal muscle mass and HGS above 42 kg compared with other groups. Low fat intake can be a result of low daily energy intake. Thus, monitoring fat intake may be important. In the current study, multiple regression analyses indicated that among the dietary nutrient intakes considered, energy (kcal/day), CHO (energy%), and fat (g/day) accounted for the variance in ALM/h^2^ and energy (kcal/day), CHO (energy%), protein (g/day), and fat (energy%) explained the variance in HGS.

We found an inverse association between total omega-3 fatty acids (g/day) and muscle strength. This finding does not strengthen the emerging hypothesis that the intake of total omega-3 fatty acids is positively associated with muscle strength in older men [37]. This discrepancy might be due to differences in the techniques used to assess muscle strength. The relationship between the intake of omega-3 fatty acids and sarcopenia, as reflected by direct measures of muscle mass and strength, needs further investigation. In the current study, no differences in either vitamins or minerals among the muscle mass and strength groups were observed. Although vitamin D is the most researched vitamin that has been hypothesized to play a role in sarcopenia, there is currently little evidence to link the dietary intake of vitamin D with sarcopenia [50,51]. Pharmacological doses of vitamin D have been used in many interventional studies concerning sarcopenia [49]. Further, vitamin D status is typically assessed as 25-hydroxy vitamin D (25(OH)D) in blood, as it reflects the sum of vitamin D from dietary intake and sunlight exposure. In general, the association between dietary micronutrients and muscle mass and strength may be stronger than measured by the FFQ. More work is warranted to elucidate the potential benefits of micronutrient intake to prevent sarcopenia and support healthy aging.

Low muscle mass among study participants was more prevalent when classifying participants into low and normal muscle mass groups based on the local cutoff of ALM/h^2^ compared with the international one. We found that neither daily energy intake nor nutrient intake differed significantly between muscle mass groups when considering the international cutoff. This might be explained by the relatively small number of participants in the low muscle mass group when defined by the international cutoff.

The association between increasing age and sarcopenia has been well established. The same association between age and muscle mass and strength was observed in this study. We also found that height, body weight, and BMI were positively associated with muscle mass and strength. This result is in line with a previous study carried out in older adults [50]. The associations between muscle mass (kg) and ALM/h^2^ or HGS in this study were unsurprising, given the fact that sarcopenia is characterized by loss of muscle mass and strength. In addition to the loss of muscle mass and strength, sarcopenia can be characterized by an increase in fat mass, which has been defined as sarcopenic obesity [52]. In the present study, however, lower fat mass (kg) was associated with lower ALM/h^2^. If energy intake is inadequate to meet requirements, muscle and fat are catabolized to provide energy [42].

In the current study, nutrient intake was assessed using a self-administered FFQ. FFQ is a common dietary assessment approach because it is easy to apply, retrospective (i.e., capturing usual intake over an extended period of time), and relatively cheap. However, this method has some limitations, including that the food lists are not comprehensive and are highly reliant on memory and conceptual skills. Despite of these limitations, it has been reported that applying an FFQ approach to assess the nutrient intake of healthy older adults might be applicable [53]. No strong evidence exists that older adults provide less valid self-reports using FFQs compared with younger adults [53].

Body composition including muscle mass was estimated in our study by BIA technique. This technique measures muscle mass indirectly based on whole-body electrical conductivity. Previous studies that validate the accuracy of BIA against dual-energy x-ray absorptiometry (DEXA) as reference standards have demonstrated contradictory findings. However, according to EWGSOP [4,5], BIA can be considered as a portable alternative to DEXA. The current study has several strengths. To the best of our knowledge, this was the first study to investigate the associations between dietary nutrient intake and sarcopenia, muscle mass, and strength in Arab men. This is particularly important because most sarcopenia studies are from eastern Asia and further sarcopenia research in western Asia has been recommended [54]. Moreover, noticeable muscle mass and strength declines may occur as early as 45 years of age [9]. Therefore, this study examined sarcopenia progression in adults aged 18 years and above. However, like all studies, this study has some limitations. No causal relationship can be obtained from this study because of its cross-sectional design. Nutrient intake was evaluated by FFQs over the previous 12 months and this may be subject to recall bias. Hence, measurement errors may attenuate associations between intakes and outcome measurements. Only the nutrients that were reported to be associated with muscle mass and strength in previous studies were examined in this study. The results refer only to nutrient intake from food sources; however, dietary supplements may have been consumed. We did not measure the biochemical nutrients in the blood, which are effective markers to evaluate the nutrient status. Finally, some covariables were not included in the analyses (such as physical activity that can influence muscle mass and strength).

## 5. Conclusions

Individuals with normal muscle mass and high HGS have greater daily energy, protein, and fat intake and a lower percentage of energy from CHO compared with sarcopenic participants. Our findings highlight the potentially important role of energy intake and composition as well as macronutrient intake in sarcopenia and healthy aging. Manipulating CHO, fat, and protein intakes may ameliorate the progression of sarcopenia with age. Further work is needed to improve our understanding of the effects of whole dietary nutrient intake on sarcopenia among both adult men and adult women. 

## Figures and Tables

**Table 1:** General characteristics and nutrient intake of study participants ^1^.

Parameters	Total (*n* = 441)
Age (year)	46.80 ± 15.98
Height (cm)	168.40 ± 6.90
Body weight (kg)	81.12 ± 15.55
BMI (kg/m^2^)	28.59 ± 5.08
WC (cm)	92.59 ± 20.46
Body fat (%)	27.41 ± 7.45
Fat mass (kg)	23.41 ± 9.81
Muscle mass (kg)	55.08 ± 8.00
ALM/h^2^ (kg/m^2^)	8.86 ± 1.32
HGS (kg)	39.31 ± 8.91
Energy (kcal/day)	2327.84 ± 679.59
CHO (g/day)	293.03 ± 80.57
CHO (energy%)	51.47 ± 11.46
Protein (g/day)	140.14 ± 104.64
Protein (energy%)	22.62 ± 11.07
Fat (g/day)	66.31 ± 31.79
Fat (energy%)	25.90 ± 10.09
Total omega-3 fatty acids (g/day)	0.10 ± 0.07
Vitamin D (ug/day)	2.89 ± 2.04
Vitamin E (mg/day)	3.53 ± 2.22
Ca (mg/day)	393.67 ± 175.80
Mg (mg/day)	66.72 ± 37.77

^1^ Data are presented as mean ± SD. Abbreviations: BMI, body mass index; WC, waist circumference; ALM, appendicular lean mass; HGS, handgrip strength; CHO, carbohydrate; Ca, calcium; Mg, magnesium.

**Table 2:** General characteristics and nutrient intake of study participants based on different ALM/h^2^ reference values ^1^.

Parameters	EWGSOP2			−1 SD < Reference Values Arab Men		
	Normal (ALM/h^2^ ≥ 7.0 kg/m^2^)	Low (ALM/h^2^ < 7.0 kg/m^2^)	*p*-Value ^2^	Normal (ALM/h^2^ ≥ 8.68 kg/m^2^)	Low (ALM/h^2^ < 8.68 kg/m^2^)	*p*-Value ^2^
*n* (441)	420	21		238	203	
Age (year)	49 (32–60)	62 (47–67.5)	0.002	42.91 ± 14.46	51.37 ± 16.49	<0.001
Height (cm)	168.45 ± 6.92	167.39 ± 6.51	0.493	169.21 ± 6.98	167.45 ± 6.70	0.008
Body weight (kg)	81.98 ± 15.22	63.93 ± 11.79	<0.001	89.04 ± 14.89	71.84 ± 10.30	<0.001
BMI (kg/m^2^)	28.87 ± 4.94	22.90 ± 4.37	<0.001	31.08 ± 4.77	25.66 ± 3.68	<0.001
WC (cm)	95.5 (85–105)	92 (76–98)	0.090	95.81 ± 20.86	88.78 ± 19.26	<0.001
Body fat (%)	27.41 ± 7.18	27.48 ± 11.85	0.979	28.46 ± 6.86	26.18 ± 7.93	<0.001
Fat mass (kg)	23.36 ± 9.69	18.75 ± 11.44	0.035	26.31 ± 10.15	19.42 ± 7.95	<0.001
Muscle mass (kg)	55.69 ± 7.65	42.74 ± 3.74	<0.001	59.73 ± 6.33	49.62 ± 6.08	<0.001
HGS (kg)	39.75 ± 8.76	30.57 ± 7.29	0.003	42.35 ± 8.15	35.68 ± 8.41	<0.001
Energy (kcal/day)	2322.71 ± 680.47	2430.39 ± 669.55	0.479	2415.16 ± 713.87	2225.46 ± 623.35	0.003
CHO (g/day)	291.82 ± 80.37	317.23 ± 82.84	0.159	290.16 ± 79.52	296.40 ± 81.85	0.418
CHO (energy%)	51.39 ± 11.48	53.14 ± 11.11	0.493	49.27 ± 11.35	54.05 ± 11.06	<0.001
Protein (g/day)	139.60 ± 104.32	150.94 ± 113.12	0.629	151.63 ± 113.82	126.67 ± 91.19	0.011
Protein (energy%)	22.62 ± 11.09	22.67 ± 11.52	0.985	23.58 ± 11.71	21.50 ± 10.24	0.047
Fat (g/day)	66.33 ± 32.05	61.97 ± 26.47	0.540	72.00 ± 34.80	59.24 ± 26.32	<0.001
Fat (energy%)	25 (18–32)	19 (16–28)	0.214	27.11 ± 10.29	24.47 ± 9.71	0.006
Total omega-3 fatty acids (g/day)	0.10 ± 0.07	0.10 ± 0.05	0.899	0.10 ± 0.07	0.10 ± 0.07	0.336
Vitamin D (ug/day)	2.86 ± 2.02	3.39 ± 2.41	0.247	2.80 ± 2.04	2.99 ± 2.04	0.314
Vitamin E (mg/day)	3.25 (1.66–4.89)	3.48 (2.15–4.69)	0.427	3.37 ± 2.30	3.73 ± 2.1	0.093
Ca (mg/day)	396.57 ± 177.51	335.76 ± 126.77	0.122	399.37 ± 184.58	386.99 ± 165.12	0.462
Mg (mg/day)	55.09 (41.24–81.17)	45.67 (40.33–97.38)	0.725	68.09 ± 38.12	65.11 ± 37.38	0.410

^1^ Data are presented as mean ± SD or median (Q1–Q3). ^2^ *p*-value significant < 0.05. *p*-value tested by unpaired *t*-test to compare mean differences or nonparametric Mann–Whitney U to compare median differences. Abbreviations: EWGSOP2, Second European Working Group on Sarcopenia in Older People; BMI, body mass index; WC, waist circumference; ALM, appendicular lean mass; HGS, handgrip strength; CHO, carbohydrate; Ca, calcium; Mg, magnesium.

**Table 3:** General characteristics and nutrient intake of study participants based on different HGS reference values ^1^.

Parameters	HGS (kg)			*p*-Value ^2^
	<27 kg	27–42 kg	>42 kg	
*n* (432)	35	237	160	
Age (year)	63.49 ± 14.76(ab) ^3^	48.84 ± 15.51(ac)	40.11 ± 13.12(bc)	<0.001
Height (cm)	164.06 ± 5.80(a)	166.64 ± 6.37(b)	172.01 ± 6.25(ab)	<0.001
Body weight (kg)	71.83 ± 12.69(ab)	78.33 ± 13.81(ac)	87.28 ± 16.34(bc)	<0.001
BMI (kg/m^2^)	88.06 ± 25.02	93.06 ± 19.55	93.06 ± 20.76	0.384
WC (cm)	26.79 ± 5.04(a)	28.21 ± 4.72(b)	29.51 ± 5.37(ab)	0.004
Body fat (%)	27.96 ± 8.80	27.90 ± 7.15	26.44 ± 7.44	0.139
Fat mass (kg)	20.79 ± 9.40	22.61 ± 9.03	24.33 ± 10.72	0.079
Muscle mass (kg)	48.86 ± 7.34(ab)	52.96 ± 7.22(ac)	59.67 ± 7.05(bc)	<0.001
ALM/h^2^ (kg/m^2^)	7.73 ± 1.16(ab)	8.6578 ± 1.22(ac)	9.4188 ± 1.27(bc)	<0.001
Energy (kcal/day)	2259.76 ± 535.90	2252.57 ± 612.69(a)	2467.23 ± 787.04(a)	<0.001
CHO (g/day)	291.42 ± 81.12	290.74 ± 77.68	297.69 ± 84.45	0.693
CHO (energy%)	52.17 ± 11.56	52.69 ± 11.12(a)	49.49 ± 11.71(a)	0.022
Protein (g/day)	130.84 ± 93.82	130.29 ± 92.48(a)	159.16 ± 122.36(a)	0.023
Protein (energy%)	21.77 ± 10.50	22.03 ± 10.87	23.89 ± 11.56	0.230
Fat (g/day)	63.41 ± 27.57	63.16 ± 32.67(a)	71.09± 31.26(a)	0.046
Fat (energy%)	26.14 ± 11.07	25.27 ± 10.22	26.57 ± 9.52	0.442
Total omega-3 fatty acids (g/day)	0.13 ± 0.14(ab)	0.01 ± 0.06(ac)	0.10 ± 0.06(bc)	0.018
Vitamin D (ug/day)	3.33 ± 2.09	3.01 ± 2.15	2.69 ± 1.87	0.141
Vitamin E (mg/day)	3.56 ± 1.82	3.39 ± 2.03	3.71 ± 2.57	0.383
Ca (mg/day)	424.21 ± 155.74	395.38 ± 169.80	383.66 ± 183.80	0.444
Mg (mg/day)	67.68 ± 40.75	65.67 ± 37.46	67.40 ± 37.33	0.886

^1^ Data presented as mean ± SD. ^2^ *p*-value significant < 0.05, tested by one-way ANOVA. ^3^ The same letter for two groups means that there is a significant difference using Tukey’s post hoc test. Abbreviations: HGS, handgrip strength; BMI, body mass index; WC, waist circumference; ALM, appendicular lean mass; CHO, carbohydrate; Ca, calcium; Mg, magnesium.

**Table 4:** Pearson’s correlation coefficient of ALM/h^2^ and HGS with general characteristics and nutrient intake.

Parameters	ALM/h^2^ (*n* = 441)		HGS (kg) (*n* = 432)	
	Pearson Correlation	*p*-Value ^1^	Pearson Correlation	*p*-Value
Age (year)	−0.31	<0.001	−0.45	<0.001
Height (cm)	0.11	0.018	0.44	<0.001
Body weight (kg)	0.74	<0.001	0.37	<0.001
BMI (kg/m^2^)	0.28	<0.001	0.05	0.338
WC (cm)	0.74	<0.001	0.19	<0.001
Body fat (%)	0.23	<0.001	−0.12	0.016
Fat mass (kg)	0.50	<0.001	0.12	0.017
Muscle mass (kg)	0.80	<0.001	0.53	<0.001
Energy (kcal/day)	0.17	<0.001	0.12	0.014
CHO (g/day)	−0.04	0.419	0.01	0.829
CHO (energy%)	−0.24	<0.001	−0.13	0.007
Protein (g/day)	0.18	<0.001	0.12	0.009
Protein (energy%)	0.17	<0.001	0.09	0.040
Fat (g/day)	0.18	<0.001	0.09	0.075
Fat (energy%)	0.07	0.117	0.04	0.445
Total omega-3 fatty acids (g/day)	−0.02	0.622	−0.09	0.041
Vitamin D (ug/day)	−0.05	0.258	−0.12	0.015
Vitamin E (mg/day)	−0.08	0.102	0.07	0.177
Ca (mg/day)	0.03	0.565	−0.04	0.388
Mg (mg/day)	0.05	0.323	0.00	0.988

^1^*p*-value significant < 0.05. Abbreviations: ALM, appendicular lean mass; HGS, handgrip strength; BMI, body mass index; WC, waist circumference; CHO, carbohydrate; Ca, calcium; Mg, magnesium.

## Data Availability

The datasets used in the current study are available from the corresponding author on reasonable request.
